# Supplementary material for: Transcriptomic Insights into Mechanisms of Early Seed Maturation in the Garden Pea (Pisum sativum L.)
Source: Cells. 2020 Mar 23;9(3):779. doi: 10.3390/cells9030779 (PMC7140803; doi:10.3390/cells9030779)

Sprint−2(20)

Global Summary

%DE = 0.81

genes with fdr < 0.2 = 1543 (473 + / 1070 −)

genes with fdr < 0.1 = 1443 (430 + / 1013 −)

genes with fdr < 0.05 = 1267 (347 + / 920 −)

genes with fdr < 0.01 = 861 (165 + / 696 −)

genes in genesets = 0

<FC> = 0

<t−score> = −34.55

<p−value> = 0.01

<fdr> = 0.19

Portrait

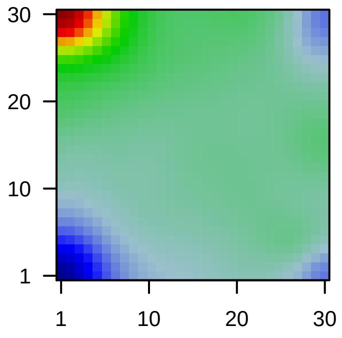

Top 100 DE genes

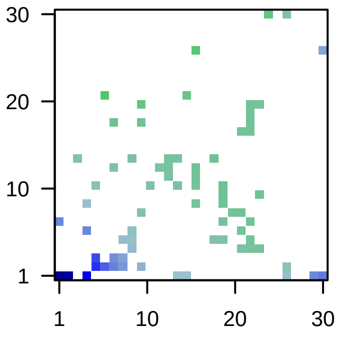

Global Genelist

Rank	ID	log(FC)	p−value	fdr	Metagene	Description
1	comp15269_c	−0.08	1e−13	1e−09	4 x 9	
2	comp4838_c0	−0.03	3e−12	2e−07	7 x 13	
3	TRINITY_DN3	−0.02	4e−10	3e−07	21 x 6	
4	TRINITY_DN8	−0.39	1e−09	3e−07	30 x 1	
5	TRINITY_DN1	−0.22	2e−09	3e−07	4 x 1	
6	TRINITY_DN2	−1.15	2e−09	4e−07	1 x 1	
7	TRINITY_DN1	−0.14	4e−09	4e−07	1 x 7	
8	TRINITY_DN1	−0.13	4e−09	6e−07	24 x 30	
9	TRINITY_DN2	−0.11	7e−09	6e−07	22 x 17	
10	TRINITY_DN6	−0.56	7e−09	1e−06	1 x 1	
11	TRINITY_DN7	−0.48	1e−08	1e−06	30 x 1	
12	TRINITY_DN7	−0.03	1e−08	1e−06	11 x 11	
13	TRINITY_DN2	−0.58	2e−08	1e−06	1 x 1	
14	TRINITY_DN2	−0.55	2e−08	1e−06	1 x 1	
15	TRINITY_DN7	−0.02	2e−08	1e−06	21 x 6	
16	TRINITY_DN2	−0.04	2e−08	3e−06	16 x 26	
17	TRINITY_DN1	−0.32	3e−08	3e−06	30 x 1	
18	TRINITY_DN1	−0.21	5e−08	3e−06	30 x 1	
19	TRINITY_DN2	−0.01	5e−08	3e−06	21 x 8	
20	comp6342_c0	−0.26	5e−08	3e−06	4 x 1	

p−values

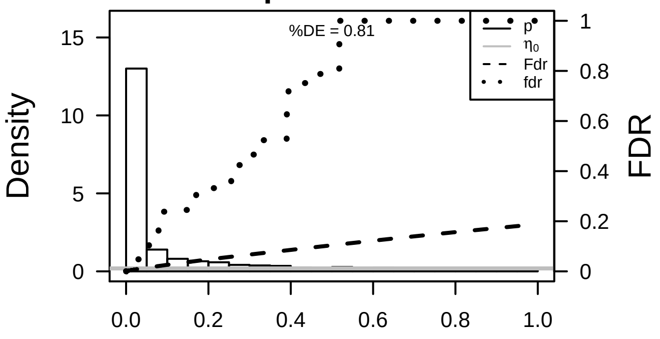

Supplement: Supplementary file 1 [file cells-09-00779-s001.zip › Supplementary materials/FileS4.zip!/FileS3/TFs - Results/Summary Sheets - Groups/Reports/Sprint.2.20..pdf]
